# The positive expression of genotype VII Newcastle disease virus (Malaysian isolate) in Japanese quails (*Coturnix coturnix japonica*)

**DOI:** 10.14202/vetworld.2017.542-548

**Published:** 2017-05-25

**Authors:** Lizma Felisha Mazlan, Noor Farhana Bachek, Siti Nor Azizah Mahamud, Lokman Hakim Idris, Tan Sheau Wei, Abdul Rahman Omar, Mohd Hezmee Mohd Noor

**Affiliations:** Department of Veterinary Preclinical Sciences, Faculty of Veterinary Medicine, Universiti Putra Malaysia, 43400 Serdang Selangor, Malaysia

**Keywords:** infections, intraocular, Japanese quails, Newcastle disease virus

## Abstract

**Aim:**

Genotype VII Newcastle disease virus (NDV) is the most predominant NDV strains that circulating in Malaysia; thus, this study was aimed to determine the susceptibility of Japanese quails toward genotype VII NDV. Clinical signs, gross pathological lesions of organs, positive detection of virus in organs and cloacal swabs, as well as the expression of the antibody titer, were used as parameters to assess the susceptibility of Japanese quails following infection of genotype VII NDV.

**Materials and Methods:**

About 20 quails were divided into three groups (n=8 for Groups A and B; n=4 for the control group). The quails in the Groups A and B were infected via intraocular route with 0.03 ml of 103.5 ELD50 and 107.0 ELD50 of NDV strain IBS 002, respectively, while the control group received 1× phosphate-buffered saline. Cloacal swabs and necropsy were taken on day 7 post-infection for all quails were subjected to one-step reverse transcription real-time quantitative polymerase chain reaction (RT-qPCR) for detection of virus and examination for gross pathological lesion, respectively. Blood serums of infected quails were taken on day 10, 14, and 21 post-day infections and were subjected for hemagglutination inhibition (HI) assay.

**Results:**

Depression and ruffled feathers, trachea rales, leg paralysis, and torticollis were shown in some of the quails in both infected groups. Based on statistical analysis, there was no significant difference (p>0.05) in clinical signs between the infected groups. The results for RT-qPCR were found to be negative for all groups, and no gross pathological lesions of organs observed for quails in both infected groups. Trachea, proventriculus, and cecal tonsil were taken for the detection of NDV by RT-qPCR, and some of the organ samples showed positive detection of virus in both infected groups. HI assay showed an increase in mean titers of antibody across time and between infected groups.

**Conclusion:**

In summary, Japanese quails are susceptible to genotype VII NDV based on parameters assessed.

## Introduction

Newcastle disease virus (NDV) or avian paramyxovirus type 1 is a non-segmented, single-stranded, negative-sense RNA virus which is a member of the genus Avulavirus of the family Paramyxoviridae. The virus widespread among wild and domestic birds with all bird species and some other vertebrates including humans (transitory conjunctivitis) is susceptible to be infected [1].

In general, strains of NDV are classified as velogenic, mesogenic, and lentogenic [2]. NDV strains are grouped into different genotypes and based on the sequence and phylogenetic analysis of the F gene; NDV strains are classified into Class I or II. Strains of Class I are avirulent in chickens, whereas strains in Class II are subdivided into at least 18 genotypes from I to XVIII [3].

Limited information is available regarding the prevalence of Newcastle disease (ND) in quail industry in Malaysia. In some studies obtained from other countries, Lima *et al*. [4] found quails as important carriers of ND virus. On the other hand, Higgins and Wong [5] and Higgins [6] stated that quails are rather resistant to NDV infection, but they may become infected under stress conditions. However, quails were found to be susceptible to natural infection with a velogenic strain of ND virus [4,7]. In view of the increasing interest in quail farming by many potential farmers in Malaysia, relevant information about the susceptibility of the Japanese quails against ND should be considered. Nowadays, genotype VII NDV is the predominant virus circulating in Asia including Malaysia and numerous geographical regions, such as Europe, China, Middle East, and South Africa [8]. Therefore, a preliminary experimental infection of Japanese quails particularly with genotype VII ND was, therefore, conducted to clarify the actual role played by the quails (*Coturnix coturnix japonica*) in the epidemiology of ND in Malaysia.

## Materials and Methods

### Ethical approval

Handling of the quails was conducted in accordance with the laboratory animal care guidelines, and the study protocol was approved by the IACUC at the Faculty of Veterinary Medicine, UPM (reference no. UPM/IACUC/FYP.2015/FPV.032).

### Experimental design

The quails were divided into three groups, which were eight quails for each of both Groups A and B, and four quails for the control group. Quails in the Groups A and B were separately placed at different levels of a double storey cage, in a room with controlled environment while quails in the control group were located in the different room with a controlled environment. All the quails were given commercialized feed based on corn and soybean meal and water *ad-libitum*.

Before the virus inoculation, serum collected from the quails from all groups was subjected for hemagglutination inhibition (HI) assays to ensure the quails were free from NDV infection. Inoculation of IBS 002 strain of genotype VII NDV was done via intraocular route; quails in Group A were given 103.5 ELD50 and quails in Group B were given 107.0 ELD50. Quails in the control group received 0.03 ml of 1× sterile phosphate buffered saline (PBS) via the intraocular route. Clinical lesions in all quails after the inoculation of NDV were recorded. Cloacal swab sampling was done on 7 days post-infection (dpi) for all quails from both infected groups and the control group for detection of NDV by reverse transcriptase real-time quantitative polymerase chain reaction (RT-qPCR). Necropsy was conducted on the same day, 7 dpi, for halves of the quails from each group to examine the gross lesions of the organs following experimental infection with NDV (n=4 for each of both Groups A and B; n=2 for control group). Organs, such as the trachea, proventriculus, and cecal tonsil, were taken for the detection of NDV by RT-qPCR. The remaining quails (n=4 for each of both Groups A and B; n=2 for control group) were kept for blood collection to obtain the serum for the measurement of antibody against NDV. Blood was collected at basilic vein on 10 dpi, 14 dpi, and 21 dpi. After third blood collection was performed on 21 dpi, all quails were euthanized through cervical dislocation method.

### Quails and management

About 20 Japanese quails (*C. coturnix japonica*) of 1 week of age were purchased from Puyumas Farmbest Sdn. Bhd., Malaysia. The quails were raised according to standard procedures in an open house system before being transferred to Universiti Putra Malaysia (UPM). For the NDV challenge study, the quails were transferred to Experimental Animal House at Faculty of Veterinary Medicine, UPM. The Japanese quails were housed in cages, with water and feed offered *ad-libitum*. The diet was based on corn and soybean meal.

### Genotype VII NDV

The isolate IBS 002 strain was obtained from the Laboratory of Vaccine and Immunotherapeutics, Institute of Bioscience (IBS), UPM. The strain was first isolated in 2011 from a vaccinated broiler farm in Johor, Peninsular Malaysia. Based on sequence analysis of the F cleavage site, the virus is confirmed to be velogenic (Acc. No. JQ809695). The virus is further classified as a genotype VII velogenic NDV based on a phylogenetic study based on the F gene, detection of multiple basic amino acids on the F protein cleavage site, intracerebral pathogenicity index of 1.76, and the mean death time of 51.2 h [8]. Virus with concentrations of 103.5 ELD50/0.03ml and 107.0 ELD50/0.03 ml were prepared.

### Observation of clinical signs

The clinical signs of quails were observed twice daily from 1 dpi until 21 dpi to observe if there were clinical lesions manifested by Japanese quails following infection of IBS 002 strain. The clinical signs that were expected to be shown by quails were based on research studies conducted by Roohani *et al*. [9] and Rasoli *et al*. [10]; clinical signs of infected chickens with IBS 002 strain include diarrhea, trachea rales, depression and ruffled feathers, loss of appetite, mortalities, and neurological signs.

### Gross pathological lesions of organs

Necropsy of quails was performed on 7 dpi involving four birds from both Groups A and B and two birds from the control group. Observation of gross post-mortem lesions in quails is important to prove that infection of NDV will produce significant lesions signs toward quails. The gross pathological lesions of organs that were expected to be shown by quails infected with IBS 002 were referred to gross pathological lesions of organs of chicken that have been infected with velogenic NDV. Lesions such as congestion and hemorrhages at muscle, trachea, and visceral organs such as proventriculus, intestine, cecal tonsil, and spleen were expected to be seen in infected quails.

### Serology testing

A total of 37 serum samples were collected including eight blood samples prior experimental infection for screening purpose, 10 blood samples (n=4 for each of both Groups A and B; n=2 for control group) on 10 dpi, 10 blood samples (n=4 for each of both Groups A and B; n=2 for control group) on 14 dpi and nine blood samples (n=4 for Group A, n=3 for Group B; n=2 for control group) on 21 dpi. Blood was collected from the basilic vein using 29G of 1 ml syringe. Before the procedure, collection site was cleaned and disinfected using 70% ethanol in the form of alcohol swabs (Heinz Herenz Hamburg/Germany). The collected blood was placed in a sterile plain test tube with a rubber stopper and labeled accordingly. The blood collected in plain tubes was centrifuged at 5000 rpm for 15 min to obtain the serum. Hemagglutination (HA) and HI assays were performed using the standard microtiter plate method as recommended by the OIE [11]. The HI tests were carried out with 4 HA units of NDV reference strains per well.

### Detection of NDV

#### Detection of NDV in cloacal swab specimens

Cloacal swab sampling was done on 7 dpi from eight quails in each of both Groups A and B, and four quails from the control group. Sterile collection swab was used for swabbing, and the swab was immersed in 1 ml of 1× sterile PBS solution. Cloacal swab specimens were kept in −80°C for later use. Viral RNA was later extracted using an RNeasyPlus Mini Kit (Qiagen, Germany) according to the manufacturer’s instructions.

#### Detection of NDVin organ samples

Trachea, proventriculus, and cecal tonsil were collected on 7 dpi after the cloacal swabs were carried out. The necropsy was performed from four quails for each of both Groups A and B and two quails from the control group. In summary, each sample was minced using a pair of scissors and then transferred into an individual sterile tube containing 3 ml of 1× PBS. The suspension was then homogenized using a homogenizer (TissueRuptor^®^, Qiagen, Germany) and kept in −80°C for later use. The viral RNA was later extracted using TRIzol^®^ RNA Isolation Reagents (Invitrogen, USA) according to the manufacturer’s instructions.

#### Measurement of RNA concentration

After the RNA extraction, 2 µl of the aliquot was transferred into a Cuvette G1.0 (Eppendorf, Germany) using a micropipette (Eppendorf, Germany) and placed in a BioSpectrometer^®^ (Eppendorf, Germany). For quantitating the amount of RNA, readings were taken at the wavelengths of 260 and 280 nm. An OD of 1 corresponds to 40 µg/ml for single-stranded RNA. The ratio between the readings at 260 and 280 nm (OD 260: OD 280) provides an estimate of the purity of the nucleic acid. Pure preparations of RNA have OD 260: OD 280 values of 1.8 and 2.0, respectively.

#### One-step RT-qPCR

One-step RT-qPCR is used to detect the presence or absence of the virus in specimens and samples of the infected quails. Detection of NDV was performed for 20 cloacal swab specimens (n=8 for each of both Groups A and B; n=4 for control group) and 30 organ samples (n=12 for each of both Groups A and B; n=6 for control group). Extracted RNA was subjected to RT-qPCR by iTaq Universal probes One-step Kit (Bio-Rad, USA) and processed based on manufacturer’s instruction. The master mix reactions were prepared and all the components were scaled proportionally according to sample number and reaction volumes. In RT-qPCR, NDV velogenic IBS 002 isolate was used as the positive control. Nuclease-free water was used for no template control (NTC). Positive control and NTC were included to ensure the reaction has been set up correctly with no contamination of reagents or foreign RNA.

The amplification was performed using CFX96™ Real-Time system (Bio-Rad, USA). The optimized cycling conditions of the one-step RT-qPCR were carried out as recommended by Rasoli *et al*. [10] as described in Table-1. Data analysis was performed based on CFX™ Manager Software (Bio-Rad, USA).

**Table-1:** The primers and probe used in the one-step RT-qPCR.

Primer/Probe	Sequence	Position on F gene
Probe	5’(FAM)-AAGCGTTTCTGTCTCCTTCCTCCA-(BHQ)3’	396-373
Forward	5’TCCGCAAGATCCAAGGGTCT3’	342-361
Reverse	5’ CGCTGTTGCAACCCCAAG3’	442-425

RT-qPCR: Reverse transcription real-time quantitative polymerase chain reaction

### Statistical analysis

The statistical analysis was performed using the computer package GraphPad Prism, version 5.0. Test of independence, Chi-square was performed to measure the association of different challenge dose of virus toward the clinical signs shown by quails. Antibody titers were expressed as a mean±standard deviation of Log2 HI titer. Next, analysis of variance (Two-way ANOVA) was performed to measure the significant differences of antibody reaction across time and between groups. Differences were considered significant at alpha=0.05.

## Results

### Clinical signs

**Figure-1:**
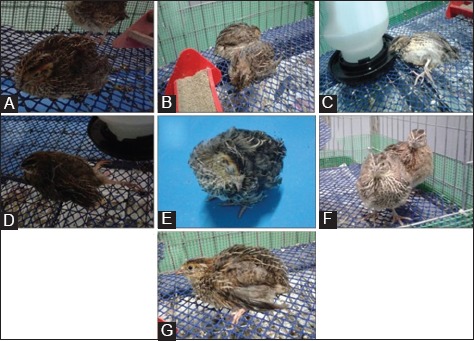
The observed clinical signs of Newcastle disease in the infected quails from 7 dpi onward. Panel A: A quail in the Group B showed ruffled feathers and depression; Panel B: Mild depression and ruffled feathers in quails from the Group A; Panel C: Leg paralysis observed on 13 dpi in quail from the Group A; Panel D: Leg paralysis was showed in a quail from the Group B on 16 dpi; Panel E: Torticollis was observed on 14 dpi in a quail from the Group B; Panel F: Quails in the control group were bright and alerts; Panel G: A quail from the control group with bright eyes.

**Figure-2:**
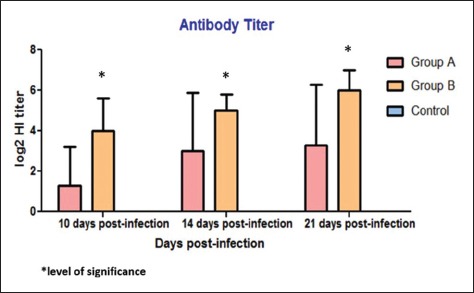
Expression of antibody across time and dose of infection based on two-way of ANOVA.

Clinical signs of ND in quails were observed started from 7 dpi. Clinical signs shown include depression and ruffled feathers, trachea rales, leg paralysis, and torticollis. Depression and ruffled feathers were observed for all quails in the infected group started from 7 dpi and markedly seen on 10 dpi (Figure-1). Depression and ruffled feathers were prominently expressed in quails from the Group B (Figure-1, Panel A) as compared to quails in the Group A (Figure-1, Panel B). Trachea rales started to be observed on 10 dpi with one quail in the Group A and two quails in the Group B. One quail from the Group A showed leg paralysis on 13 dpi (Figure-1, Panel C) whereas leg paralysis was observed on 16 dpi in quail from the Group B (Figure-1, Panel D). Torticollis was observed in one quail from the Group B on 14 dpi (Figure-1, Panel E), and the quail was found dead on 16 dpi. All quails in the control group were bright and alert (Figure-1, Panels F and G). The frequency distribution of clinical signs shown by quails following infection of genotype VII NDV was shown in Figure-2. Based on statistical analysis of Chi-square test (GraphPad Prism 5), there was no significant difference (p>0.05) of association of differently challenged dose of virus toward the clinical signs shown by quails between Groups A and B. All quails in the infected group showed clinical signs of ND regardless of virus infection dosage.

### Gross pathological lesions of organs

Quails in the control group that is given 1× PBS did not show any gross pathological lesions upon necropsy on 7 dpi. The result is also similar with quails in infected groups as there were no gross pathological lesions of organs were seen in quails for both Groups A and B following infection of velogenic genotype VII NDV in quails.

### Serology testing

The screening was done in the representatives of quails before infection of genotype VII NDV and quails showed serologically negative for ND by HI assays. Seroconversions were detected in all surviving quails in both Groups A and B; detected on 10 dpi, then start to increase at 14 dpi and continued in increasing till the end of observation at 21 dpi. All the data were expressed as a mean±standard deviation of Log2 HI titer (Table-2). The data were analyzed by GraphPad Prism 5.0, to measure the extent of antibody reaction across time and challenged dose. Based on statistical analysis of two-way ANOVA, there was no significant difference (p>0.05) of antibody reaction across time; however, the antibody reaction was significantly difference (p<0.05) between Groups A and B (Figure-2).

**Table-2:** Log2 HI titer at 10, 14 and 21 days postinfection.

Days post infection	Group A*						Group B**						Control group***			
		
	Log2 HI Titer				Mean±SD	Positive ratio	Log2 HI Titer				Mean±SD	Positive ratio	Log2 HI Titer		Mean±SD	Positive ratio
		
	A1	A2	A3	A4			B1	B2	B3	B4			C1	C2		
10	1	4	N/D	N/D	1.3±1.9	2/4	2	4	4	6	4.0±1.6	4/4	N/D	N/D	-	0/2
14	3	7	N/D	2	3.0±2.9	3/4	4	5	5	6	5.0±0.8	4/4	N/D	N/D	-	0/2
21	4	7	N/D	2	3.3±3.0	3/4	-	6	5	7	6.0±1.0	3/4	N/D	N/D	-	0/2

* Four quails were used as sample size in Group A (n=4) on 10, 14 and 21 days postinfection.

** Four quails were used as sample size in Group B (n=4) on 10 and 14 days postinfection. Only three quails were used as sample size on 21 days postinfection (one of the quail [B1] was found dead on 16 days postinfection due to torticollis).

*** Two quails were used as sample size in Control Group (n=2) on 10, 14 and 21 days postinfection. N/D=Not detected, SD=Standard deviation, HI: Hemagglutination inhibition

### Detection of NDV in cloacal swab specimens

Cloacal swab sampling was done for all the quails from both infected groups and the control group (n=20). The RT-qPCR assay showed there was no amplification curve of cloacal swab specimens detected. All the generated data for RT-qPCR were tabulated (Table-3).

**Table-3:** Detection of virus in cloacal swab and organ samples by RT-qPCR.

Type of sample	Fluorescent*	Target	Sample name**	Detection of virus***
Cloacal swab	FAM	Velogenic	A1	−
	FAM	Velogenic	A2	−
	FAM	Velogenic	A3	−
	FAM	Velogenic	A4	−
	FAM	Velogenic	A5	−
	FAM	Velogenic	A6	−
	FAM	Velogenic	A7	−
	FAM	Velogenic	A8	−
	FAM	Velogenic	B1	−
	FAM	Velogenic	B2	−
	FAM	Velogenic	B3	−
	FAM	Velogenic	B4	−
	FAM	Velogenic	B5	−
	FAM	Velogenic	B6	−
	FAM	Velogenic	B7	−
	FAM	Velogenic	B8	−
	FAM	Velogenic	C1	−
	FAM	Velogenic	C2	−
	FAM	Velogenic	C3	−
	FAM	Velogenic	C4	−
	FAM	Velogenic	NDV reference strain	+
	FAM	Velogenic	NDV reference strain	+
	FAM	Velogenic	NTC	−
Organ	FAM	Velogenic	A1: CT	−
	FAM	Velogenic	A1: P	−
	FAM	Velogenic	A1: T	−
	FAM	Velogenic	A2: CT	−
	FAM	Velogenic	A2: P	−
	FAM	Velogenic	A2: T	−
	FAM	Velogenic	A3: CT	−
	FAM	Velogenic	A3: P	−
	FAM	Velogenic	A3: T	−
	FAM	Velogenic	A4: CT	+
	FAM	Velogenic	A4: P	−
	FAM	Velogenic	A4: T	−
	FAM	Velogenic	B1: CT	−
	FAM	Velogenic	B1: P	−
	FAM	Velogenic	B1: T	−
	FAM	Velogenic	B2: CT	+
	FAM	Velogenic	B2: P	-
	FAM	Velogenic	B2: T	−
	FAM	Velogenic	B3: CT	+
	FAM	Velogenic	B3: P	−
	FAM	Velogenic	B3: T	+
	FAM	Velogenic	B4: CT	+
	FAM	Velogenic	B4: P	−
	FAM	Velogenic	B4: T	+
	FAM	Velogenic	NDV reference strain	+
	FAM	Velogenic	NDV reference strain	+
	FAM	Velogenic	NTC	−

* FAM=Fluorescent reporter dye 5-carboxyfluorescein.

** A1-A8=8 quails from Group A, B1-B8=8 quails from Group B, C1-C4=4 quails from control group.

*** Positive (+) amplification showed quantification cycle, Cq value ranging from 20 to 35 cycles. Negative (−) or no amplification is represented by quantification cycle, Cq value>35 cycles. NTC=No template control, RT-qPCR: Reverse transcription real-time quantitative polymerase chain reaction, NDV: Newcastle disease virus

### Detection of NDV in organ samples

From the total of eight infected quails and two quails from the control group that was sacrificed for this study, 30 organ samples such as the trachea, proventriculus, and cecal tonsil were processed for PCR assay. The RT-qPCR assay showed six organ samples were positive for NDV (Table-3). Amplification curves of organ samples that represent positive detection are shown for Quail A4, B2, B3, and B4. Out of the eight infected quails that were necropsied on 7 dpi, four were found to be positive with detection of genotype VII NDV by RT-qPCR (50%). In summary, one out of four quails (25%) from Group A showed positive virus detection. Three out of four quails (75%) from Group B showed positive virus detection.

## Discussion

The susceptibility of Japanese quails toward genotype VII NDV was determined in this study by assessing parameters such as clinical signs, gross pathological lesions of organs, virus detection in organs and cloacal swabs by one-step RT-qPCR and expression of antibody titer by HI assay. Two doses were used in this experimental design, 103.5 ELD50 of NDV strain IBS 002 and 107.0ELD50 of NDV strain IBS 002. The purpose of using the low dose of 103.5ELD50 NDV strain IBS 002 is to have a general idea regarding the minimum infection dose required to indicate the susceptibility of quails toward velogenic genotype VII NDV. Surprisingly, some of the quails in the group that have been infected with the low dose, 103.5 ELD50 NDV strain IBS 002 still showed clinical signs, detection of virus in organ sample and antibody formation against NDV.

Quails in both groups which were infected with genotype NDV IBS 002 strain showed depression and ruffled feathers, trachea rales, and neurological signs such as leg paralysis. Depression and ruffled feathers were started on 7 dpi and markedly seen on 10 dpi, trachea rales were noticed started on 10 dpi and leg paralysis was observed started on 13 dpi. A quail that was infected with high-dose titer of virus, 107.0 ELD50 NDV strains IBS 002 showed torticollis and died on 16 dpi. The clinical signs vary with the pathogenicity of the isolate and the species of bird [10]. Rasoli *et al.*, [10] reported chickens infected with IBS 002 showed diarrhea, trachea rales, depression ruffled, and loss of appetite and mortalities. The variation in the manifestation of clinical signs was observed when comparing between species. Clinical signs associated with the various strains can be different in species other than chickens [10].

The velogenic pathotype is divided into a neurotropic form, which has respiratory and neurologic signs, and a viscerotropic form with hemorrhagic intestinal lesions [11]. This classification is not always that clear and many strains have various manifestations in different birds [10]. Quails in both infected groups showed no gross pathological lesions of organs following infection of velogenic genotype VII NDV. The finding is similar to an experimental study conducted by Mohamed *et al.*, [13], where Japanese quails infected with velogenic NDV did not show overt gross lesions. The author hypothesized that NDV undergo limited replication and persisted for a short period in tissue, or it replicated at very low level in quails.

Organs, such as cecal tonsil and trachea, were targeted for the detection of virus by RT-qPCR in this study due to the fact that trachea is the main tissue tropism of NDV [13]. The cecal tonsil which is a lymphoid organ that favors the replication of virus was claimed to be one of the tissue tropism of NDV [14]. Attempts to detect NDV from different organs such as brain, spleen, and intestine could be considered in the future as some studies proved chicken infected with NDV strain had exhibited lesion to visceral organs which suggesting there was initially occurrence of viral replication [12]. There was a high occurrence of detection of virus in quails given a higher infection dose suggesting that extensive of replication of virus occurred in organs, thus making the detection is much possible. The cloacal swab was done on 7 dpi for detection of NDV by RT-qPCR. RNA extracted from swab showed lower yield and concentration as compared to organs. There was no attempt to isolate the virus by egg inoculation as the negative detection of virus in cloacal swabs due to the low replication of NDV in quails as mentioned by Mohamed *et al.*, [13]. RT-qPCR was done in this study as it offers a reliable tool for quick detection of NDV, and this was agreed by Mazumder *et al.*, 2012, which has performed the RT-PCR in his study for NDV detection although the gold standard is to perform virus isolation and serological identification followed by *in vivo* pathogenicity testing [8].

Antibodies against NDV can be detected approximately after 6-10 days [15]. From 10 dpi to 21 dpi, almost all the quails in both Groups A and B exhibited positive HI titers for NDV. Based on the statistical analysis (GraphPad Prism 5) of two-way ANOVA, there was no significant difference (p>0.05) of antibody reaction across time; however, the antibody reaction was significantly different (p<0.05) between Groups A and B. Extent of antibody reaction was significantly different between groups and suggests that extensive virus replication occur in quails given with high virus titer and led to significant high production of antibody as high virus and/or virus-infected cells can stimulate B lymphocytes to produce significant higher amount of antibody [16,17].

## Conclusion

Japanese quails showed clinical signs, detection of virus in organs and expression in the antibody titer thereby confirming the susceptibility of Japanese quails toward infection of genotype VII NDV.

## Authors’ Contributions

LFM and SNAM carried out the experiments as part of their final year project under the guidance of NFB for the laboratory works. LHI and MHMN designed the experiment and prepared the first draft of the manuscript. TSW and ARO designed and assisted the molecular works. All authors read and approved the final manuscript.

## References

[1] Leighton F.A, Heckert R.A, Thomas N.J, Hunter D.B, Atkinson C.T (2007). Newcastle disease and related avian paramyxoviruses. Infectious Diseases of Wild Birds.

[2] Alexander D.J, Senne D.A, Saif Y.M, Fadly A.M, Glisson J.R, McDougald L.R, Nolan K, Swayne D.E (2008). Newcastle disease, other avian paramyx-oviruses, and pneumovirus infections. Diseases of Poultry.

[3] Snoeck C.J, Owoade A.A, Couacy-Hymann E, Alkali B.R, Okwen M.P, Adeyanju A.T, Komoyo G.F, Nakouné E, LeFaou A, Muller C.P (2013). High genetic diversity of Newcastle disease virus in poultry in West and Central Africa: Cocirculation of genotype XIV and newly defined genotypes XVII and XVIII. J. Clin. Microbiol.

[4] Lima F.S, Santin E, Paulillo A.C, Doretto L, De Moraes V.R.M, Schocken R.P (2004). Japanese quail (*Coturnix coturnix japonica*) as Newcastle disease virus carrier. Int. J. Poult. Sci.

[5] Higgins D.A, Wong F.S (1968). Newcastle disease in a flock of Japanese quails. Vet Rec.

[6] Higgins D.A (1971). Nine disease outbreaks associated with myxoviruses among ducks in Hong Kong. Trop Anim. Health Prod.

[7] Czirják G.Á, Köbölkuti L.B, Cadar D, Ungvári A, Niculae M, Bolfă P (2007). An outbreak of the Newcastle disease in Japanese quail (*Coturnix coturnix japonica*). Bull. USAMV-CN.

[8] Mazumder A.C, Khatun S, Nooruzzaman M, Chowdhury E.H, Das P.M, Islam M.R (2012). Isolation and identification of Newcastle disease viruses from field outbreaks in chickens and pigeons. Bangladesh Vet.

[9] Roohani K, Tan S.W, Yeap S.K, Ideris A, Bejo M.H, Omar A.R (2015). Characterisation of genotype VII Newcastle disease virus (NDV) isolated from NDV vaccinated chickens, and the efficacy of LaSota and recombinant genotype VII vaccines against challenge with velogenic NDV. J. Vet. Sci.

[10] Rasoli M, Yeap S.K, Tan S.W, Moeini H, Ideris A, Bejo M.H, Omar A.R (2014). Alteration in lymphocyte responses, cytokine and chemokine profiles in chickens infected with genotype VII and VIII velogenic Newcastle disease virus. Comp. Immunol. Microbiol. Infect. Dis.

[11] OIE (2012). Manual of Diagnostic Test and Vaccines for Terrestial Animals. Chapter 2.3.14. Newcastle disease (infection with Newcastle disease virus.

[12] United States Department of Agriculture (2016). Newcastle disease.

[13] Mohamed M.A, Hafez M.S.A (2016). The susceptibility of Japanese quails to the infection with chicken originated Newcastle disease virus. J Adv. Vet. Res.

[14] Levy R, Spira G, Zakay-Rones Z (1975). Newcastle disease virus pathogenesis in the respiratory tract of local or systemic immunized chicken. Avian Dis.

[15] Samuel A, Nayak B, Paldurai A, Xiao S, Aplogan G.L, Awoume K.A, Webby R.J, Ducatez M.F, Collins P.L, Samal S.K (2013). Phylogenetic and pathotypic characterization of Newcastle disease viruses circulating in West Africa and efficacy of a current vaccine. J. Clin. Microbiol.

[16] Miller P.J, Afonso C.L, ElAttrache J, Dorsey K.M, Courtney S.C, Guo Z, Kapczynski D.R (2013). Effects of Newcastle disease virus vaccine antibodies on the shedding and transmission of challenge viruses. Dev. Comp. Immunol.

[17] Denman A.M (1979). Lymphocyte function and virus infections. J Clin. Pathol.

